# Activation of CREB‐mediated autophagy by thioperamide ameliorates β‐amyloid pathology and cognition in Alzheimer’s disease

**DOI:** 10.1111/acel.13333

**Published:** 2021-03-08

**Authors:** Jiangong Wang, Bin Liu, Yong Xu, Meizi Yang, Chaoyun Wang, Mengmeng Song, Jing Liu, Wentao Wang, Jingjing You, Fengjiao Sun, Dan Wang, Dunjiang Liu, Haijing Yan

**Affiliations:** ^1^ Department of Pharmacology College of Basic Medicine Binzhou Medical University Yantai China; ^2^ Institute for Metabolic and Neuropsychiatric Disorders Binzhou Medical University Hospital Binzhou China; ^3^ Department of Thyroid Breast Surgery Dongying People’s Hospital Dongying China

**Keywords:** Alzheimer’s disease, autophagy, cognitive dysfunction, cyclic AMP response element‐binding protein, histamine, histamine H3 receptor, lysosome, neuronal loss, β‐amyloid, β‐secretase 1

## Abstract

Alzheimer's disease (AD) is an age‐related neurodegenerative disease, and the imbalance between production and clearance of β‐amyloid (Aβ) is involved in its pathogenesis. Autophagy is an intracellular degradation pathway whereby leads to removal of aggregated proteins, up‐regulation of which may be a plausible therapeutic strategy for the treatment of AD. Histamine H3 receptor (H3R) is a presynaptic autoreceptor regulating histamine release via negative feedback way. Our previous study showed that thioperamide, as an antagonist of H3R, enhances autophagy and protects against ischemic injury. However, the effect of thioperamide on autophagic function and Aβ pathology in AD remains unknown. In this study, we found that thioperamide promoted cognitive function, ameliorated neuronal loss, and Aβ pathology in APP/PS1 transgenic (Tg) mice. Interestingly, thioperamide up‐regulated autophagic level and lysosomal function both in APP/PS1 Tg mice and in primary neurons under Aβ‐induced injury. The neuroprotection by thioperamide against AD was reversed by 3‐MA, inhibitor of autophagy, and siRNA of *Atg7*, key autophagic‐related gene. Furthermore, inhibition of activity of CREB, H3R downstream signaling, by H89 reversed the effect of thioperamide on promoted cell viability, activated autophagic flux, and increased autophagic‐lysosomal proteins expression, including Atg7, TFEB, and LAMP1, suggesting a CREB‐dependent autophagic activation by thioperamide in AD. Taken together, these results suggested that H3R antagonist thioperamide improved cognitive impairment in APP/PS1 Tg mice via modulation of the CREB‐mediated autophagy and lysosomal pathway, which contributed to Aβ clearance. This study uncovered a novel mechanism involving autophagic regulating behind the therapeutic effect of thioperamide in AD.

## Graphical abstract

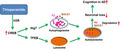

## 1 INTRODUCTION

Alzheimer's disease (AD) is an age‐related, progressive neurodegenerative disease which pathologically characterized by the presence of extracellular amyloid‐β (Aβ)‐containing senile plaques and intracellular hyperphosphorylated tau‐containing neurofibrillary tangles, neuroinflammation, synaptic loss and neuronal death, neocortical atrophy and the progressive deterioration of cognitive function(Yankner & Lu, 2009; Crews & Masliah, 2010; Keskin et al., 2017; Long & Holtzman, 2019). The deposition and impaired clearance of extracellular Aβ is one of the primary causes of AD (Li et al., 2007; Mawuenyega et al., 2010; Sinha & Lieberburg, 1999). Therefore, promoting Aβ clearance is becoming one of effective treatment for AD (Boland et al., 2018; Li et al., 2007; Luo et al., 2020; Sevigny et al., 2017).

Autophagy is an intracellular degradation pathway whereby unwanted cytosolic contents are engulfed in double‐membrane bound vesicles and then delivered to lysosomes for digestion (Boya et al., 2013; Di Meco et al., 2020; Rubinsztein et al., 2005; Yang et al., 2005). Emerging evidence suggests that autophagy is compromised in AD, resulting in accumulation of dysfunctional organelles, misfolded protein aggregates, and neuronal death (Colacurcio et al., 2018; Hou et al., 2019; Kerr et al., 2017; Komatsu et al., 2006; Nixon, 2007; Zeng et al., 2019). Beclin 1, a protein with a key role in autophagy, decreases in brain regions of patients with AD (Pickford et al., 2008). Either heterozygous deletion or mutation of Beclin 1 in mice decreases neuronal autophagy and results in neurodegeneration and disruption of lysosomes (Esteves et al., 2019; Lee & Gao, 2008; Pickford et al., 2008; La Spada et al., 2017). Activation of autophagy decreases Aβ pathology and alleviates cognitive dysfunction in various animal models (Steele et al., 2013; Yang et al., 2011a, 2011b; Yi et al., 2020; Yu et al., 2005). Therefore, reversing the autophagic dysfunction may serve as an innovative therapeutic target for the treatment of AD (Boland et al., 2008; Di Meco et al., 2017; Orr & Oddo, 2013; Yang et al., 2011b).

Histamine is an endogenous neurotransmitter in the brain (Wada et al., 1991). Up to now, there are four subtypes of receptors have been identified: H1R, H2R, H3R, and H4R, of which H1R–H3R are found in brain (Brown et al., 2001; Haas & Panula, 2003; Haas et al., 2008). Histamine H3 receptor (H3R) is a presynaptic autoreceptor that regulates histamine release from histaminergic neurons via negative feedback way (Arrang et al., 1983; Morisset et al., 2000), as well as a heteroreceptor that regulates the release of other neurotransmitters (Clapham & Kilpatrick, 1992; Dai et al., 2007; Hansen et al., 2010; Schlicker et al., 1988, 1989, 1993). H3R is a G‐protein‐coupled receptor (GRCR) that activates Gi/o proteins to inhibit adenylyl cyclase (AC) and cAMP‐response element‐binding protein (CREB) activity(Leurs et al., 2005). A number of experiments have provided evidences that inhibition of H3R could alleviate cognitive deficit in AD (Bitner et al., 2011; Brioni et al., 2011; Medhurst et al., 2007; Patnaik et al., 2018). Therefore, it raises the possibility of H3R antagonist for AD treatment. However, the potential mechanisms remain to be clarified. Our previous study has showed that H3R antagonist activates autophagy and protects against cerebral ischemic injury (Yan et al., 2014). In addition, activation of H3R downstream signaling CREB, which is involved in improving cognitive dysfunction and Aβ pathology in AD (Wang et al., 2018; Yin et al., 2016), could also up‐regulate autophagy (Chong et al., 2018; Liu et al., 2019; Seok et al., 2014). Nevertheless, whether CREB‐mediated autophagy is involved in the alleviated cognitive deficit and Aβ pathology by H3R antagonist in AD remains undetermined.

In this study, we hypothesize that H3R antagonist may reduce the AD‐related Aβ pathology and improve cognitive dysfunction through directly up‐regulate autophagy via activating CREB pathway. We would show the beneficial effect of H3R antagonist and explain a novel mechanism that H3R antagonist‐mediated autophagy in AD.

## 2 RESULTS

### 2.1 Thioperamide rescues cognitive dysfunction in APP/PS1 Tg mice in vivo

**FIGURE 1:**
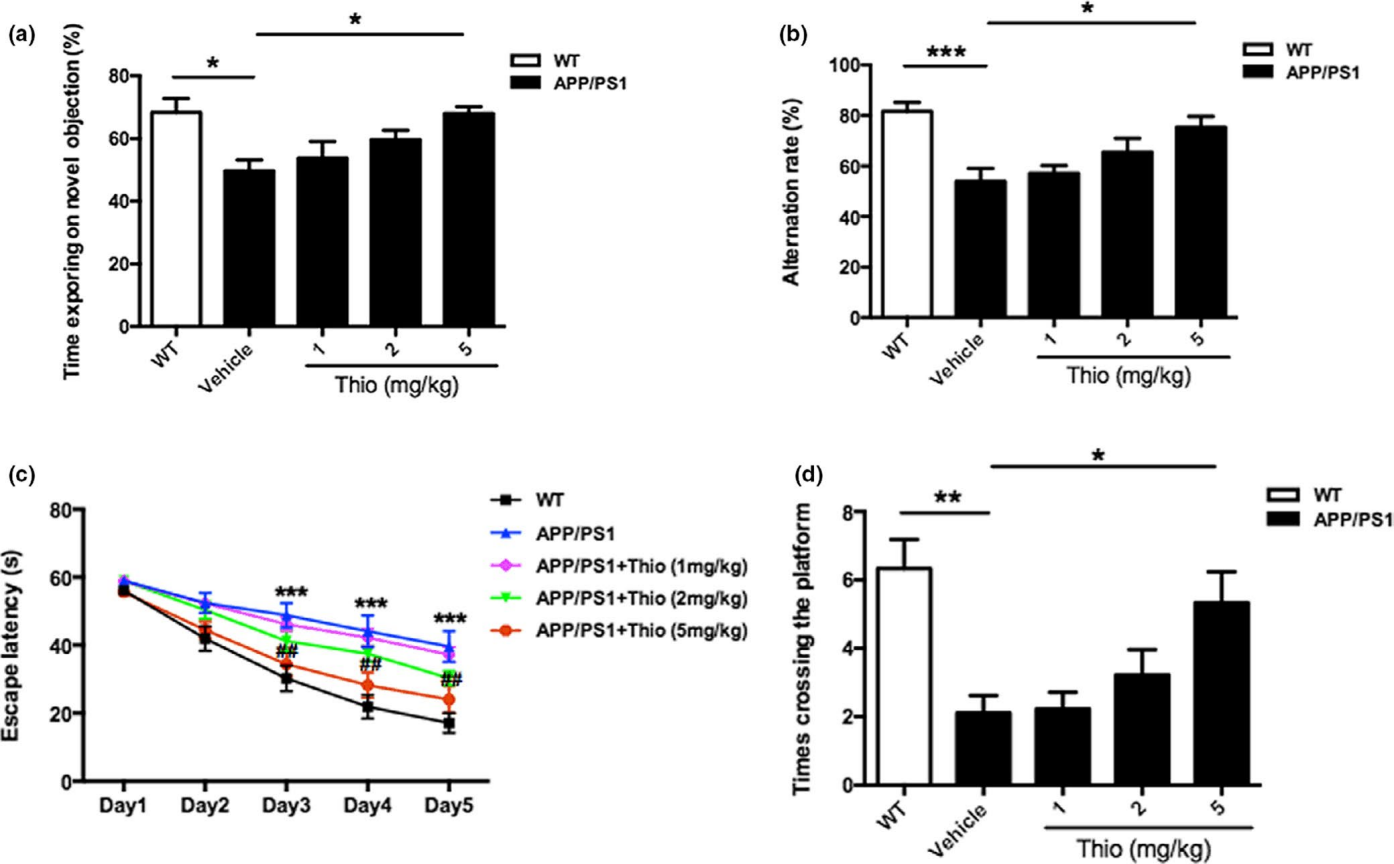
Effect of thioperamide on cognitive deficits in APP/PS1 mice *in vivo*. (a) The NOR test showing the exploring time on new object in those receiving vehicle and thioperamide (i.p., 1, 2, 5 mg/kg) in WT and APP/PS1 Tg mice. (b) The Y maze test showing the effect of thioperamide on alternation rate in WT and APP/PS1 Tg mice. (c, d) The MWM test showing the effect of thioperamide on the escape latency on training days (c) and times crossing the platform on testing day (d) in WT and APP/PS1 Tg mice. n = 9 per group. *p < 0.05, **p < 0.01, ***p < 0.001 in a, b, and d. ***p < 0.001 *vs*. the WT group; ^##^ *p* < 0.01 *vs*. the APP/PS1 group in c. Mean ± SEM. One‐way ANOVA followed by Tukey's *post hoc* (a, b, d) and two‐way ANOVA followed by Bonferroni *post hoc* test (c)

In order to investigate the role of H3R in AD, we firstly determined the expression of H3R in the brain. It was shown that H3R expressed extensively in cortex and hippocampus in mice (Figure S1). Afterward, we used 8‐month‐old APP/PS1 Tg mice which have the mutated human APPs we and PSEN1ΔE9, to examine the therapeutic efficacy of thioperamide, a H3R antagonist in AD. The effect of thioperamide on cognitive dysfunction in APP/PS1 Tg mice was tested. The novel object recognition (NOR) test indicated that time spending on novel objection decreased significantly in the APP/PS1 group compared with the WT group (from 68.36 ± 4.47% to 49.70 ± 3.44%, *p* < 0.05, Figure 1a), which was reversed significantly by administration of thioperamide (5 mg/kg) (to 68.04 ± 2.14%, *p* < 0.05, Figure 1a). In the Y maze (YM) test, we observed a decreased SA% in the APP/PS1 group compared with the WT group (from 81.63 ± 3.61% to 53.92 ± 5.11%, *p* < 0.001, Figure 1b). Administration of thioperamide increased the SA% to 75.28 ± 4.35% significantly (*p* < 0.05, Figure 1b). In morris water maze (MWM) test, the escape latency increased significantly in the APP/PS1 group on day 3 to day 5 (*p* < 0.01, Figure 1c), which was significantly reversed by administration of thioperamide (*p* < 0.01, Figure 1c). Moreover, times crossing the platform decreased in APP/PS1 Tg mice (from 6.33 ± 0.85 to 2.11 ± 0.51, *p* < 0.01, Figure 1d) on day 6, which was also reversed remarkably by administration of thioperamide (5.33 ± 0.91, *p* < 0.05, Figure 1d). Results above suggested that thioperamide improved the cognitive impairments in APP/PS1 Tg mice *in vivo*.

### 2.2 Thioperamide attenuates neuronal cell death in APP/PS1 Tg mice in vivo

**FIGURE 2:**
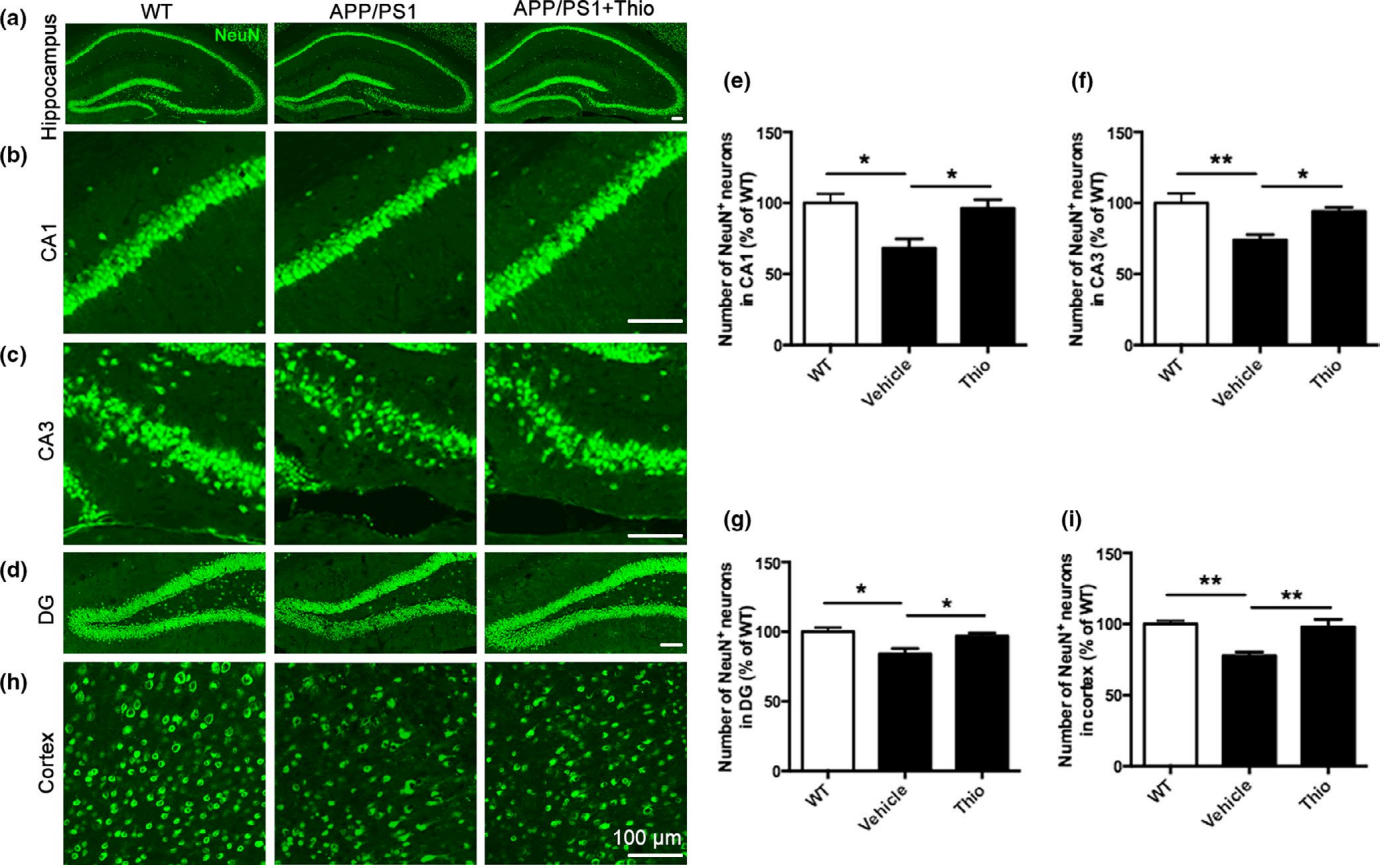
Effect of thioperamide on neuronal cell death in APP/PS1 mice *in vivo*. (a‐i) Representative immunohistochemical staining of NeuN (green) and bar graph in either hippocampus (a), including CA1 (b, e), CA3 (c, f), and DG (d, g) or cortex (h, i) showing the NeuN^+^ positive neurons in those receiving vehicle and thioperamide in WT and APP/PS1 mice. Scale bar: 100 μm. n = 5 per group. *p < 0.05, **p < 0.01. Mean ± SEM. One‐way ANOVA followed by Tukey's *post hoc* test

During the AD pathogenesis, the deposition of Aβ induces neuronal cell death, which is believed to be the main contributor for cognitive decline in AD (Hardy, 2006; Hardy & Higgins, 1992; Long & Holtzman, 2019). Therefore, we examined the neuronal death in the hippocampus and cortex by NeuN staining. Results showed that the number of NeuN^+^‐cells in the hippocampal CA1, CA3, and DG of thioperamide‐treated mice dramatically increased compared with the vehicle‐treated APP/PS1 Tg mice (from 68.30 ± 6.54% to 96.29 ± 6.15%, *p* < 0.05; from 74.08 ± 3.62% to 94.17 ± 2.85%, *p* < 0.05; from 84.02 ± 3.97% to 96.83 ± 2.23%, *p* < 0.05, respectively, Figure 2a‐g). Similarly, we also observed an increase of NeuN^+^‐cells in the cortex with administration of thioperamide compared with the vehicle‐treated APP/PS1 mice (from 77.67 ± 2.66% to 98.00 ± 5.27%, *p* < 0.01, Figure 2h,i). These results indicated that thioperamide protected against neuronal death in both hippocampus and cortex in APP/PS1 Tg mice.

### 2.3 Thioperamide induces autophagy and alleviates hippocampal and cortical amyloid pathology in APP/PS1 Tg mice in vivo

**FIGURE 3:**
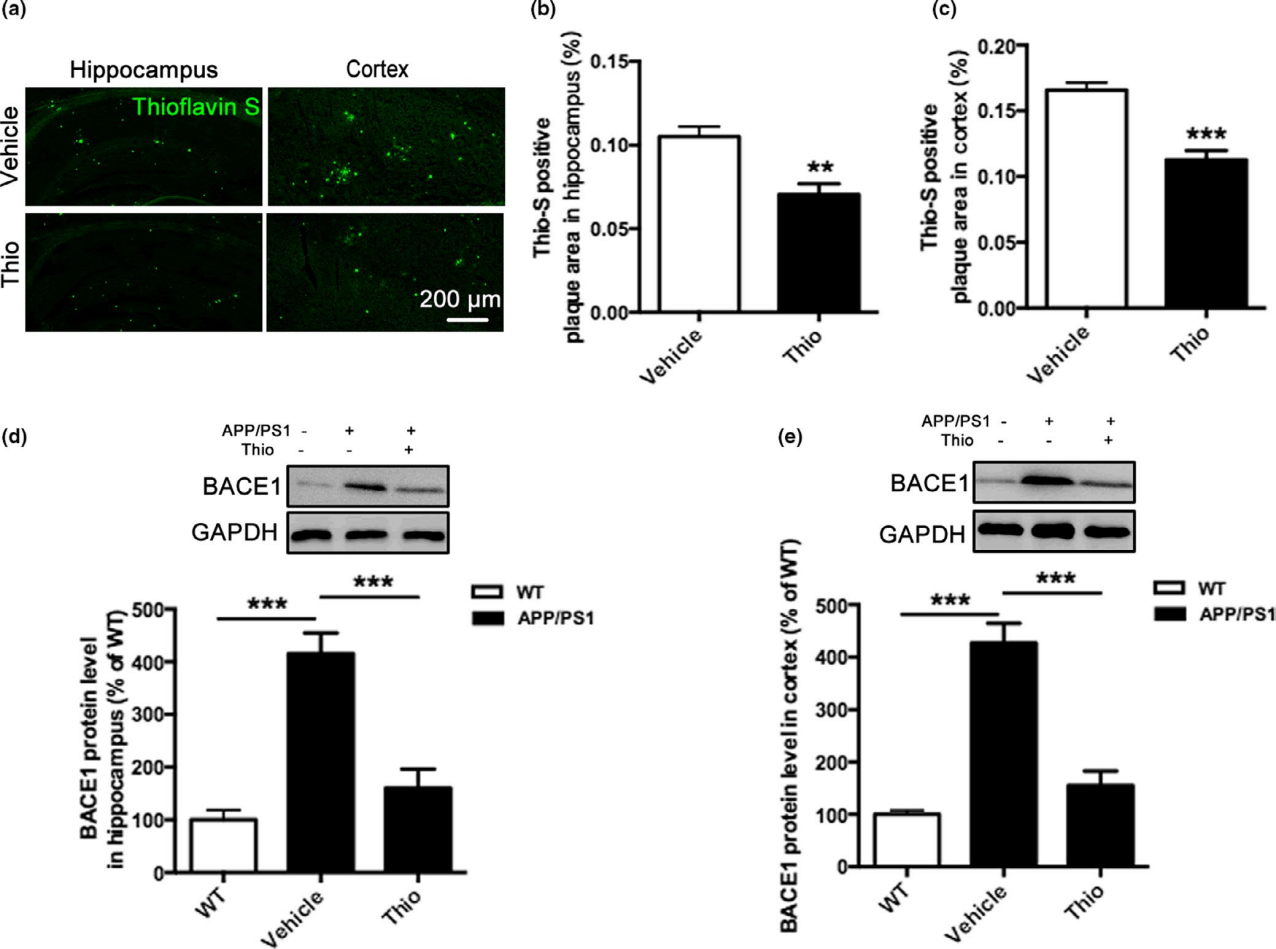
Effect of thioperamide on plaque deposition in APP/PS1 mice *in vivo*. (a‐c) Representative staining by thioflavin‐S (green) and bar graph in both hippocampus (a, b) and cortex (a, c) showing the deposition of plaque in those receiving vehicle and thioperamide in APP/PS1 mice. Scale bar: 200 μm. (d, e) Representative Western blots and bar graph showing the effect of thioperamide on BACE1 expression in hippocampus (d) and cortex (e) in WT and APP/PS1 mice. n = 4–5 per group. **p < 0.01, ***p < 0.001 *vs*. the vehicle group in b and c. ***p < 0.001 in d and e. Mean ± SEM. Student's *t* test (b, c) and one‐way ANOVA followed by Tukey's *post hoc* test (d, e)

The characterized pathological changes of AD are deposition of plaque and β‐secretase‐1 (BACE1) that is involved in Aβ accumulation (Li et al., 2007). Thus, we investigated the effect of thioperamide on thioflavin‐S‐positive plaque deposition and BACE1 expression. We observed a dramatic reduction in plaque burden in the hippocampus and cortex with thioflavin‐S staining in the thioperamide group compared with the vehicle group in APP/PS1 Tg mice (Figure 3a). The quantitative analysis indicated that thioperamide down‐regulated the area of thioflavin‐S‐positive plaque burden in both hippocampus and cortex significantly (from 0.1050 ± 0.0060% to 0.0704 ± 0.0064% in hippocampus, *p* < 0.01, Figure 3b; from 0.1658 ± 0.0056% to 0.1128 ± 0.0076% in cortex, *p* < 0.001, Figure 3c). Moreover, we investigated whether thioperamide affected the amyloidogenic pathway of Aβ production in APP/PS1 Tg mice. Western blot analysis of protein extracts showed that the levels of the BACE1 were higher in the vehicle‐treated APP/PS1 Tg mice (405.0 ± 20.75% of WT group in hippocampus, *p* < 0.001, Figure 3d; 414.9 ± 40.75% of WT group in cortex, *p* < 0.001, Figure 3e), and administration of thioperamide significantly lowered the levels of BACE1 either in hippocampus or cortex of APP/PS1 Tg mice (from 405.0 ± 20.75% to 160.8 ± 41.50% of WT group in hippocampus, *p* < 0.001, Figure 3d; from 414.9 ± 40.75% to 175.4 ± 27.75% of WT group in cortex, *p* < 0.001, Figure 3e). Additionally, we also investigated the long‐term effect of thioperamide on BACE1 inhibition. Results showed that thioperamide remarkably suppressed the expression of BACE1 in either hippocampus or cortex in APP/PS1 Tg mice 3 months after drug treatment (Figure S2). These results indicated that thioperamide significantly reduced the amyloidogenic pathway of plaque burden and Aβ production.

**FIGURE 4:**
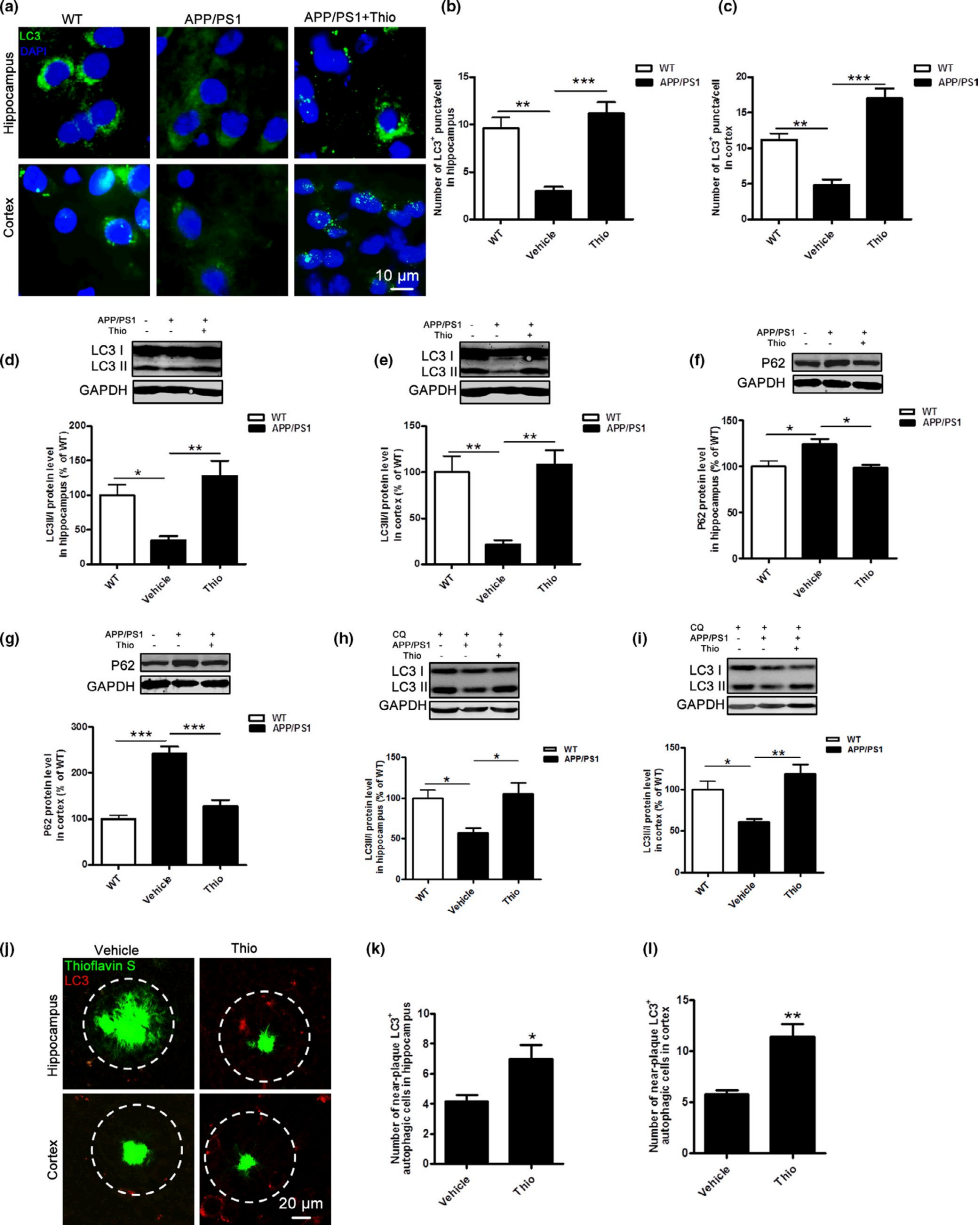
Effect of thioperamide on autophagy in APP/PS1 mice *in vivo*. (a‐c) Representative immunohistochemical staining of LC3 (green) and bar graph in both hippocampus (a, b) and cortex (a, c) showing the autophagic level in those receiving vehicle and thioperamide in WT and APP/PS1 mice. Scale bar: 10 μm. (d‐g) Representative Western blots and bar graph showing the effect of thioperamide on LC3I, LC3II (D, E), and P62 (f, g) expression in hippocampus (d, f) and cortex (e, g) in WT and APP/PS1 mice. (h, i) Representative Western blots and bar graph showing the effects of thioperamide on autophagic flux by examining the expression of LC3‐I and LC3‐II under chloroquine (10 μM) treatment. (j‐l) Representative staining of thioflavin‐S (green) along with LC3 (red) and bar graphs showing the near‐plaque autophagic cells in APP/PS1 mice with thioperamide treatment in both hippocampus (j, k) and cortex (j, l). n = 4–5 per group. *p < 0.01, **p < 0.01, ***p < 0.001 in B‐G. *p < 0.01, **p < 0.01 *vs*. the vehicle group in k and l. Mean ± SEM. One‐way ANOVA followed by Tukey's *post hoc* test (b‐i) and student's *t* test (k, l)

It is reported that autophagy is impaired in the brain tissues of AD patients and animal models (Ghavami et al., 2014; Luo et al., 2020). In order to investigate whether autophagy was involved in the reduction of amyloid plaque burden and BACE1 expression in APP/PS1 Tg mice offered by thioperamide, we examined the effect of thioperamide on the number of autophagic puncta by immunohistochemical staining and protein levels by Western blot of autophagy marker proteins. Interestingly, we found a significant increase of autophagic puncta/cell in hippocampus and cortex of APP/PS1 Tg mice with thioperamide treatment (from 31.25 ± 4.66% to 116.7 ± 12.06% of WT group in hippocampus, *p* < 0.001, Figure 4a, b; from 42.86 ± 7.14% to 151.8 ± 12.63% of WT group in cortex, *p* < 0.001, Figure 4a,c). Moreover, we also observed a significant increase of LC3II expression. The Western blot results showed that the expression of LC3II was significantly lower in hippocampus in the vehicle group in APP/PS1 Tg group compared with the WT group (reduced to 34.57 ± 6.41% of the WT group, *p* < 0.05, Figure 4d), suggesting autophagy level was declined in APP/PS1 Tg mice in hippocampus. However, the LC3II expression was dramatically higher in the thioperamide group (increased to 127.8 ± 22.09% of the WT group, *p* < 0.01, Figure 4d) compared with the vehicle group in APP/PS1 Tg mice in hippocampus. Similarly, in consistent with the autophagic level in hippocampus, we also found that thioperamide up‐regulated the expression of LC3II in cortex in APP/PS1 Tg mice (from 21.16 ± 4.91% to 108.4 ± 15.38% of the WT group, *p* < 0.01, Figure 4e), suggesting thioperamide enhanced the production of autophagy in APP/PS1 Tg mice. In addition, we examined the effect of thioperamide on P62 accumulation in APP/PS1 Tg mice. We observed a significant accumulation of P62 in either hippocampus or cortex (124.8 ± 5.29% of the WT group in hippocampus, *p* < 0.05, Figure 4f; 241.3 ± 16.08% of the WT group in cortex, *p* < 0.001, Figure 4g), which was reversed significantly by thioperamide (reduced to 98.28 ± 3.90% of the WT group in hippocampus, *p* < 0.05, Figure 4f; 127.3 ± 13.93% of the WT group in cortex, *p* < 0.001, Figure 4g) in APP/PS1 Tg mice.

To confirm the effect of thioperamide on autophagic function, we examined autophagic flux by administration of chloroquine. Interestingly, results indicated that the autophagic flux, that was revealed by LC3II expression after chloroquine treatment, decreased significantly in both hippocampus (decreased to 57.38 ± 5.71% of the WT group, *p* < 0.05, Figure 4h) and cortex (decreased to 60.81 ± 4.04% of the WT group, *p* < 0.05, Figure 4i) in APP/PS1 Tg group compared with the WT group. Thioperamide enhanced the LC3II expression in either hippocampus or cortex in APP/PS1 Tg mice (increased to 104.9 ± 13.95% of the WT group in hippocampus, *p* < 0.05, Figure 4h; increased to 119.0 ± 11.11% of the WT group in hippocampus, *p* < 0.01, Figure 4i), suggesting thioperamide enhanced the autophagic function in APP/PS1 Tg mice.

To figure out whether the effect of thioperamide on amyloid plaque clearance was relative to the enhancement of autophagy, immunofluorescence staining of LC3‐positive autophagic cells near plaque was measured in both hippocampus and cortex of APP/PS1 Tg mice. Administration of thioperamide resulted in an increased number of LC3‐positive autophagic cells around plaque with thioflavin‐S staining (from 4.200 ± 0.3742% to 7.000 ± 0.8944% in hippocampus, *p* < 0.05, Figure 4j,k; from 5.800 ± 0.3742% to 11.40 ± 1.249% in cortex, *p* < 0.01, Figure 4j,l). These results preliminarily remind us that up‐regulation of autophagy might be involved in the clearance of amyloid plaque by thioperamide in APP/PS1 Tg mice.

### 2.4 Autophagy suppression reverses the alleviated cognitive dysfunction and amyloid pathology by thioperamide in APP/PS1 Tg mice in vivo

**FIGURE 5:**
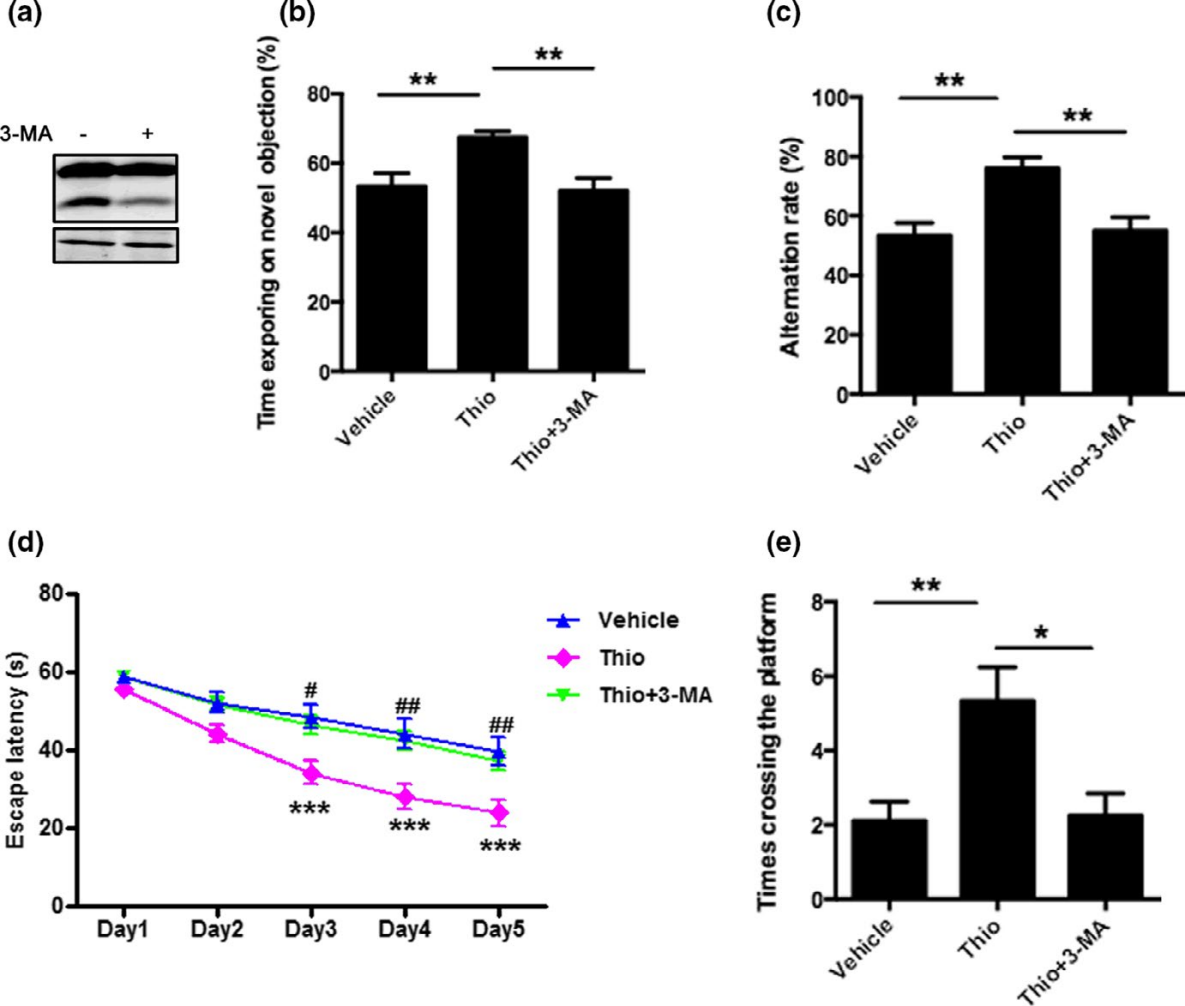
Inhibition of autophagy reverses the ameliorated cognitive dysfunction by thioperamide in APP/PS1 Tg mice *in vivo*. (a) Representative Western blots showing the expression of LC3I and LC3II after administration of 3‐MA in WT mice. (b) The NOR test showing effect of 3‐MA (i.c.v., 15 µg/mouse) and thioperamide on the exploring time on new object in APP/PS1 Tg mice. (c) The Y maze test showing the alternation rate in those receiving vehicle, thioperamide, and 3‐MA in APP/PS1 Tg mice. (d, e) The MWM test showing the escape latency on training days (d) and crossing times on testing day (e) in those receiving vehicle, thioperamide, and 3‐MA in APP/PS1 Tg mice. n = 8–11 per group. *p < 0.05, **p < 0.01 in b, c, and e. ***p < 0.001 *vs*. the vehicle group; ^#^ *p* < 0.05, ^##^ *p* < 0.01 *vs*. the thioperamide group in d. Mean ± SEM. One‐way ANOVA followed by Tukey's *post hoc* (b, c, e) and two‐way ANOVA followed by Bonferroni *post hoc* test (d)

**FIGURE 6:**
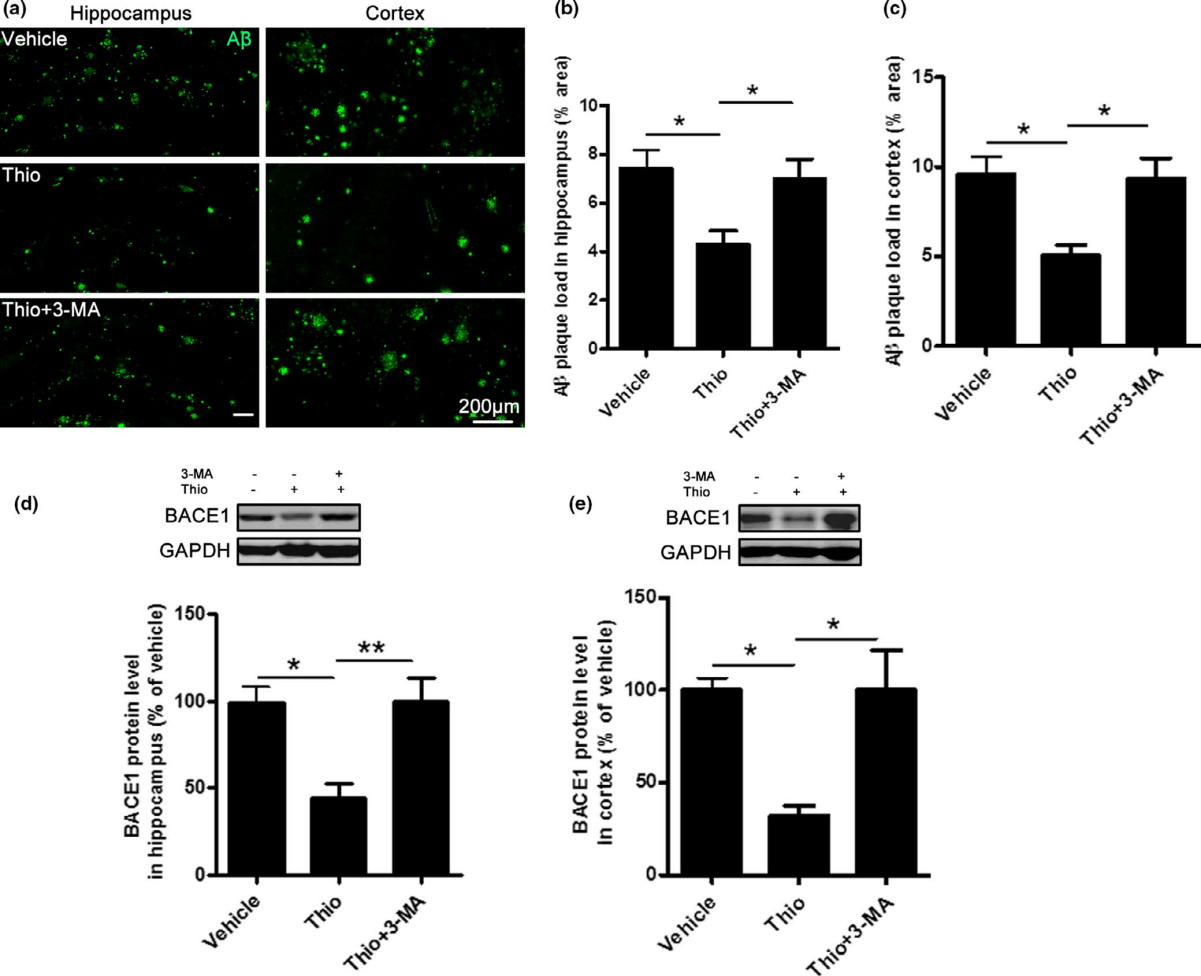
Inhibition of autophagy reverses the reduced Aβ pathology by thioperamide in APP/PS1 Tg mice *in vivo*. (a‐c) Representative immunohistochemical staining of Aβ and bar graph in both hippocampus (a, b) and cortex (a, c) showing the deposition of Aβ in those receiving vehicle, thioperamide, and 3‐MA in APP/PS1 Tg mice. Scale bar: 200 μm. (d, e) Representative Western blots and bar graph showing the effect of thioperamide and 3‐MA on BACE1 expression in hippocampus (d) and cortex (e) in APP/PS1 mice. n = 4–5 per group. *p < 0.05, **p < 0.01. Mean ± SEM. One‐way ANOVA followed by Tukey's *post hoc* test

In order to further study whether thioperamide‐induced up‐regulation of autophagy was necessary to its effects in AD, 3‐Methyladenine (3‐MA) was applied to pharmacological inhibit autophagy (Figure 5a). Results indicated that administration of 3‐MA significantly reversed the effect of thioperamide on NOR test (time spending on novel objection was reversed from 67.40 ± 1.79% to 52.09 ± 3.61%, *p* < 0.01, Figure 5b), YM test (SA% was reversed from 76.04 ± 3.72% to 55.06 ± 4.61%, *p* < 0.01, Figure 5c), and MWM test (the escape latency was reversed on day 3 to day 5, *p* < 0.05; times crossing the platform was reversed from 5.33 ± 0.91 to 2.25 ± 0.59, *p* < 0.05, Figure 5d,e). Moreover, administration of 3‐MA compromised the effect of thioperamide on Aβ clearance. The immunostaining of Aβ indicated that 3‐MA reversed either hippocampal or cortical Aβ area (from 4.32 ± 0.55% to 7.01 ± 0.80% in hippocampus, *p* < 0.05, Figure 6a,b; from 5.05 ± 0.60% to 9.37 ± 1.13% in cortex, *p* < 0.05, Figure 6a,c). The Western blot analysis showed that 3‐MA reversed the decrease of BACE1 expression by thioperamide (from 44.07 ± 8.68% to 100.2 ± 12.94% of vehicle in hippocampus, *p* < 0.01, Figure 6d; from 32.08 ± 5.35% to 100.3 ± 21.22% in cortex, *p* < 0.05, Figure 6e). Result above suggested that thioperamide improved the cognitive function and alleviated amyloidogenic pathway of Aβ burden and Aβ production through up‐regulating autophagy.

### 2.5 Thioperamide protects against Aβ‐induced injury through up‐regulation of autophagy in primary neurons in vitro

**FIGURE 7:**
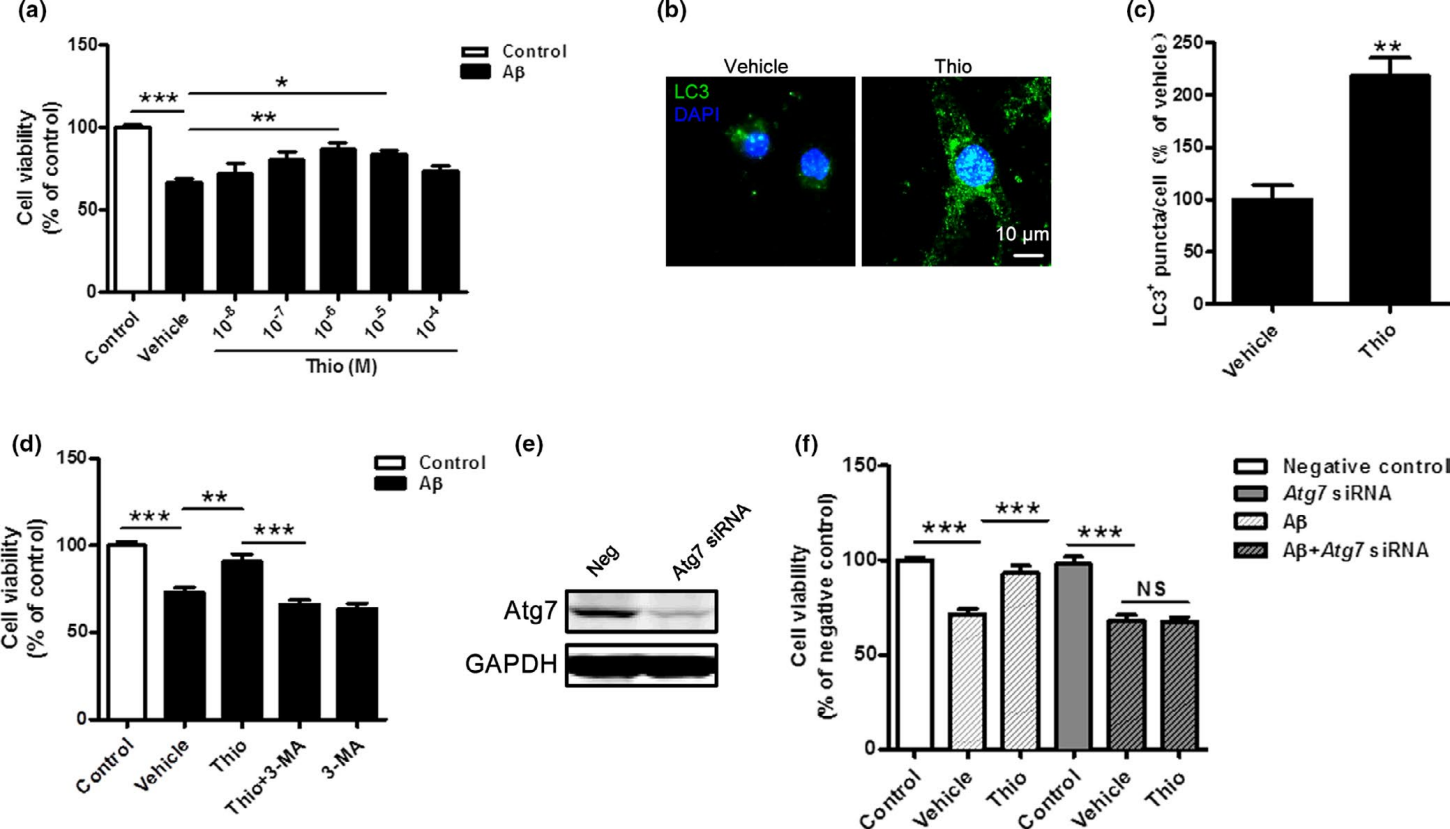
Inhibition of autophagy reverses the increased cell viability by thioperamide against Aβ‐induced injury in primary neurons *in vitro*. (a) Cell viability was tested in primary neurons with different concentrations of thioperamide (0.5 h before Aβ treatment) under Aβ‐induced (5 μM, 48 h) injury. (b, c) immunohistochemical staining of LC3 and bar graph in primary neurons showing the effect of thioperamide on autophagic puncta level under Aβ‐induced injury. Scale bar: 10 μm. (d) Cell viability was measured in those receiving thioperamide and 3‐MA (2.5 mM) under Aβ‐induced injury in primary neurons. (e) Representative Western blots showing the expression of Atg7 interfered by siRNA in primary cultured neurons. (f) Effect of thioperamide on cell viability was tested after siRNA for Atg7 and Aβ treatment in cultured primary neurons. n = 4–8 per group. *p < 0.01, **p < 0.01, ***p < 0.001 in a, d, and f. **p < 0.01 *vs*. the vehicle group in c. Mean ± SEM. One‐way ANOVA followed by Tukey's *post hoc* test (a, d, f) and student's *t* test (c)

To figure out whether thioperamide was protective against Aβ toxicity in AD, Aβ‐induced injury in primary neurons *in vitro* was used. Firstly, we determined the expression of H3R in primary cultured neurons. Results showed that H3R was highly expressed in cultured primary neurons (Figure S1). Under an incubation of 5 μM‐Aβ for 48 h, various concentrations of thioperamide on cell viability were detected by MTT. The results showed that thioperamide protected against Aβ‐induced injury in a concentration‐dependent manner. The cell viability declined to 66.26 ± 2.51% of control (*p* < 0.001, Figure 7a). Thioperamide could rescue neurons from Aβ‐induced cell viability impairment, as cell viability increased significantly to 86.87 ± 3.71% (*p* < 0.01) and 83.61 ± 2.19% (*p* < 0.05) when 10^−6^ and 10^−5 ^M of thioperamide was administrated, respectively (Figure 7a). However, no significant difference in cell viability was observed between vehicle (0 M) and lower (10^−8^ M, 10^−7^ M) or higher (10^−4^ M) concentration of thioperamide groups (*p* > 0.05, Figure 7a). Therefore, a concentration of 1 µM thioperamide is selected for the later study.

We next examined the effect of thioperamide on autophagic level under Aβ‐induced injury in primary neurons. Results showed that thioperamide significantly increased the LC3^+^ autophagic puncta (increased to 218.1 ± 17.23% of vehicle, *p* < 0.01, Figure 7b,c). Further study indicated that inhibition of autophagy by both 3‐MA and siRNA of Atg7 completely abolished the protective effect of thioperamide against Aβ‐induced injury in primary neurons. There were no differences in cell viability between the vehicle and thioperamide groups after 3‐MA administration, showing that 3‐MA alone was not neurotoxic. The increased cell viability under Aβ‐induced injury by thioperamide was observed in vehicle‐treated groups but not with 3‐MA treatment (Figure 7d). Although 3‐MA is an accepted as an autophagy inhibitor by blocking PI3 K‐class III, its non‐selective effects cannot be completely excluded. To further clarify the role of autophagy in the protection by thioperamide in AD, the key autophagy gene Atg7 was knocked down by siRNA. The silence effect is confirmed by Western blot of Atg7 (Figure 7e). The results showed that the neuroprotection by thioperamide was diminished by Atg7 knockdown (67.76 ± 3.19% with vehicle versus 67.43 ± 2.36% with thioperamide; Figure 7f). Taken together, results showed that thioperamide protected neurons against Aβ‐induced injury through up‐regulating autophagy.

### 2.6 CREB pathway is involved in the protective effect of thioperamide against Aβ‐induced injury in primary neurons in vitro

**FIGURE 8:**
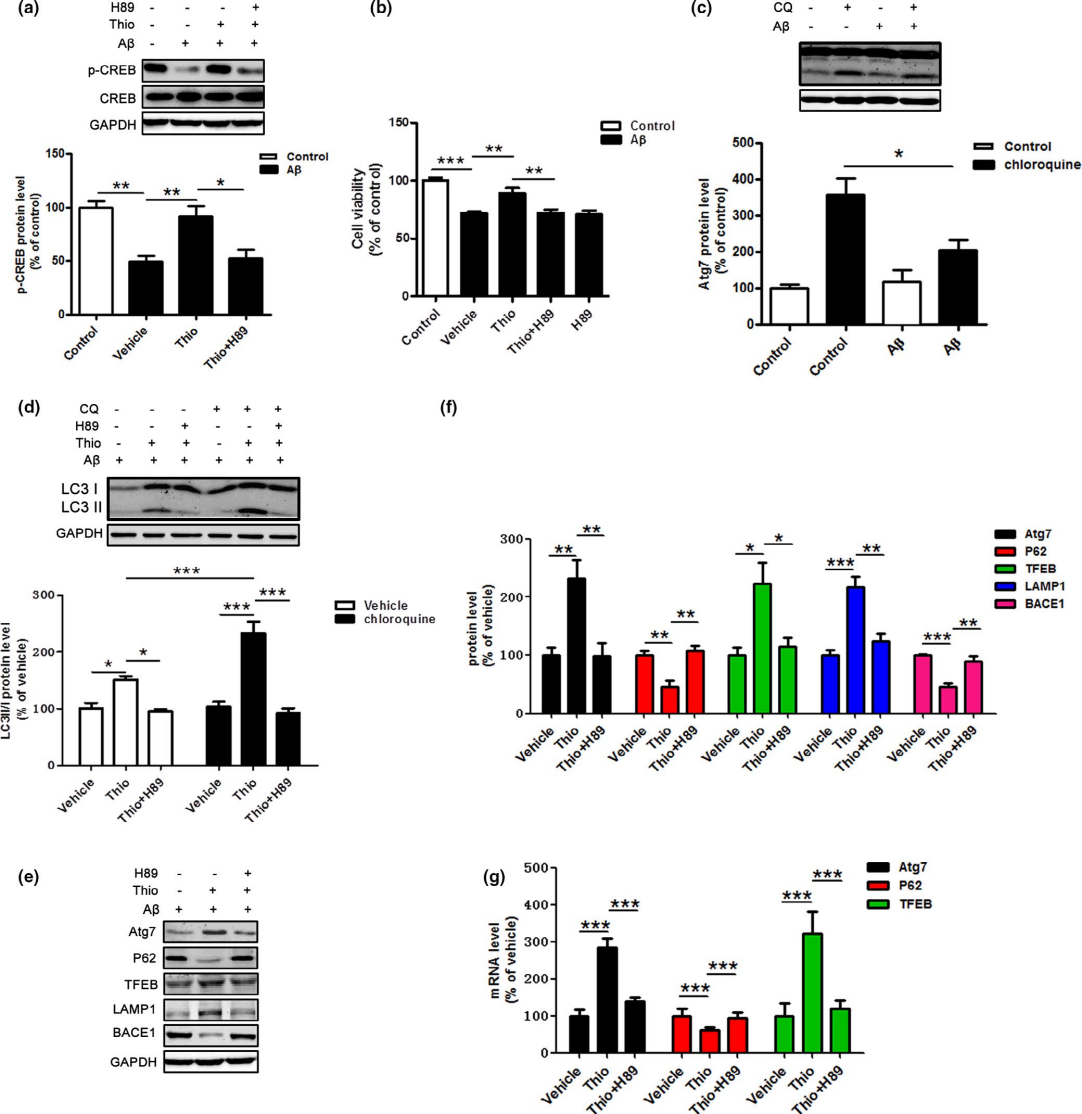
Involvement of CREB pathway in the effect of thioperamide on cell viability and autophagic activation in primary neurons *in vitro*. (a) Representative Western blots and bar graph showing the effects of thioperamide (1 μM, 0.5 h before Aβ treatment) and H89 (10 μM, 1 h before Aβ treatment) on phosphorylation of CREB after Aβ treatment in primary neurons. (b) The effects of H89 and thioperamide on cell viability under Aβ‐induced injury in primary neurons. (c) Representative Western blots and bar graph showing the effects of Aβ on expression of LC3‐I and LC3‐II on steady‐state autophagy and autophagic flux with treatment of chloroquine. (d) Representative Western blots and bar graph showing the effects of H89 and thioperamide on expression of LC3‐I and LC3‐II on steady‐state autophagy and autophagic flux with administration of chloroquine under Aβ treatment. (e) The bar graph showing the effects of H89 and thioperamide on mRNA levels of autophagic‐related proteins, including Atg7, P62, and TFEB. (f, g) Representative Western blots and bar graph showing the effects of thioperamide and H89 on expression of autophagic‐lysosomal‐related proteins, including Atg7, P62, TFEB, LAMP1, and BACE1. n = 4–7 per group. *p < 0.05, **p < 0.01, ***p < 0.001. Mean ± SEM. One‐way ANOVA followed by Tukey's *post hoc* test

The inactivation of CREB is involved in the impaired autophagy in AD mice (Chong et al., 2018). Aβ oligomers inactivate CREB in cultured neuronal cells (Zimbone et al., 2018), while its activation induces up‐regulation autophagy and alleviates Aβ‐induced injury (Singh et al., 2017; Singh, Kashyap, et al., 2017). Therefore, in order to clarify whether or not the H3R downstream CREB signaling is involved in the protective effect of thioperamide against Aβ‐induced injury, we examined the p‐CREB level. In consistent with the previous reports, decreased p‐CREB level was observed under Aβ treatment for 0.5 h (decreased to 49.51 ± 5.55% of control group, *p* < 0.01, Figure 8a). As expect, thioperamide up‐regulated the p‐CREB expression (increased to 91.81 ± 9.88% of control group, *p* < 0.01, Figure 8a), which was reversed by H89, a PKA/CREB signaling inhibitor.

In order to further investigate the involvement of CREB signaling in the protection of thioperamide on Aβ‐induced cell viability impairment in primary neurons, H89, the inhibitor of PKA/CREB was administrated to inhibit p‐CREB. The results showed that the cell viability markedly decreased in the thioperamide +H89 group compared with the thioperamide group (from 89.37 ± 4.19% to 72.24 ± 2.93% of control, *p* < 0.01, Figure 8b), suggesting an important role of CREB in the protective effect of thioperamide on Aβ‐induced injury in primary neurons. There were no differences in cell viability between the vehicle and thioperamide groups after H89 administration, showing that H89 alone was not neurotoxic. Results above revealed that the protective effect of thioperamide on Aβ‐induced injury in primary neurons might be at least in part via CREB pathway.

### 2.7 Thioperamide activates autophagic flux through CREB pathway under Aβ‐induced injury in primary neurons in vitro

Impaired lysosomal function leads to accumulation of LC3II and undigested components including Aβ in AD (Bordi et al., 2016; Guglielmotto et al., 2014; Son et al., 2012). Consistent with these results, a significant impairment of autophagic flux was observed under Aβ‐induced injury in primary neurons (from 359.1 ± 43.12% to 206.5 ± 25.78% of control, *p* < 0.05, Figure 8c). However, the level of LC3II was unchanged in the Aβ treated group compared to the control group (to 117.1 ± 34.47% of control, *p* > 0.05, Figure 8c). Moreover, we also examined autophagic flux to investigate the role of CREB in the autophagic lysosome function offered by thioperamide. Results showed that thioperamide up‐regulated the level of LC3II under administration of chloroquine (from 103.1 ± 9.17% to 233.1 ± 20.23% of vehicle, *p* < 0.001, Figure 8d), suggesting thioperamide enhanced the autophagic flux under Aβ‐induced injury. Moreover, we also found that inhibition of CREB by H89 reversed either elevated LC3II level (from 150.9 ± 6.70% to 95.05 ± 3.91% of vehicle, *p* < 0.05, Figure 8d) or augmented autophagic flux level (from 233.1 ± 20.23% to 91.79 ± 8.52% of vehicle, *p* < 0.001, Figure 8d), suggesting activation of CREB was involved in the effect of thioperamide on autophagic function.

We next studied the role of CREB in the autophagic production and lysosome function offered by thioperamide in primary neurons. Results indicated that thioperamide increased the expression of autophagic‐related protein Atg7 (increased to 232.7 ± 30.1% of vehicle, *p* < 0.01, Figure 8e,f), which was reversed by administration of H89 (reduced to 98.29 ± 22.50% of vehicle, *p* < 0.01, Figure 8e,f), indicating that activation of CREB‐mediated up‐regulation of Atg7 might be involved in the improved autophagic level by thioperamide. Inhibited accumulation of P62 by thioperamide was also compromised under administration of H89 (from 46.32 ± 10.41% to 107.4 ± 8.83% of vehicle, *p* < 0.01, Figure 8e,f), reminding us that CREB activation might be involved in autophagic regulation by thioperamide. To further clarify the mechanism of thioperamide on lysosomal function, we examined the lysosomal‐related proteins, including TFEB and LAMP1. We found that thioperamide increased the expression of both TFEB (increased to 223.6 ± 34.90% of vehicle, *p* < 0.05, Figure 8e,f) and LAMP1 (increased to 217.3 ± 17.45% of vehicle, *p* < 0.001, Figure 8e,f). Administration of H89 reversed the expression of either TFEB (reduced to 114.8 ± 15.15% of vehicle, *p* < 0.005, Figure 8e,f) or LAMP1 (reduced to 123.4 ± 13.98% of vehicle, *p* < 0.01, Figure 8e,f), suggesting that activation of CREB by thioperamide up‐regulated the expression of TFEB and LAMP1, and contributed to the improvement of lysosomal function. Additionally, the inhibited expression of BACE1 by thioperamide was also obviously reversed by administration of H89 (from 45.74 ± 6.16% to 89.46 ± 8.97% of vehicle, *p* < 0.01, Figure 8e,f), suggesting that thioperamide also suppressed BACE1 expression through activating CREB.

To further confirm the role of CREB in the autophagic‐related gene transcription, we tested the effects of H89 and thioperamide on mRNA expression of Atg7, P62, and TFEB by RT‐PCR. We found that thioperamide up‐regulated Atg7 (increased to 283.5 ± 24.94% of vehicle, *p* < 0.001, Figure 8g) and TFEB (increased to 320.9 ± 60.09% of vehicle, *p* < 0.001, Figure 8g) but suppressed P62 (decreased to 60.95 ± 7.95% of vehicle, *p* < 0.001, Figure 8g) mRNA expression compared with the vehicle group. However, either the increased mRNA expression of Atg7 (decreased to 138.0 ± 11.39% of vehicle, *p* < 0.001, Figure 8g) and TFEB (decreased to 118.9 ± 23.85% of vehicle, *p* < 0.001, Figure 8g) or the decreased mRNA level of P62 (decreased to 93.43 ± 15.72% of vehicle, *p* < 0.001, Figure 8g) was completely reversed by H89 treatment, suggesting that activation of CREB by thioperamide also regulated the transcriptional level of Atg7, P62, and TFEB.

Taken together, these results showed that thioperamide activates autophagic flux through CREB‐mediated up‐regulation of autophagic‐related protein Atg7 and lysosomal‐related protein TFEB and LAMP1.

## 3 DISCUSSION

Up to now, no therapy is available to block or slow down AD progression, and the mechanisms of AD are not fully understood (Di Meco et al., 2020). It is reported that whilse H3R is preserved in AD brain, higher density of H3R correlated with more severe dementia (Medhurst et al., 2007, 2009; Sadek et al., 2016). H3R agonist (R)‐α‐methylhistamine (RAMH) induces impaired learning and memory (Sadek et al., 2016), while H3R knockout enhanced cognitive function (Rizk et al., 2004; Toyota et al., 2002). Moreover, inhibition of H3R by antagonists alleviates cognitive deficit (Delay‐Goyet et al., 2016; Masini et al., 2017), which could be reversed by H3R agonist RAMH (Alachkar et al., 2017). Thioperamide is the most widely used experimental selective H3R antagonist which can promote the production of histamine (Liao et al., 2019). Although thioperamide also has high affinity for H4R, it is not expressed abundantly in the CNS (Witkin & Nelson, 2004). Moreover, it has also been confirmed that the H4R downstream pathways are similar to those described for H3R (Obara et al., 2020). To further explore the effect of thioperamide on AD, the *in vivo* and *in vitro* experiments were carried out by using APP/PS1 Tg mice and Aβ‐induced injury on primary neurons respectively. Firstly, we examined the expression of H3R in mice brain. H3R was expressed extensively in either hippocampus (including CA1, CA3, and DG) or cortex in mice. Moreover, H3R also expressed either in primary cultured neurons or in microglia and astrocytes. These results were consistent with the reports by Iida et al. (2015) and Mele & Juric (2013) that H3R expressed not only in neurons but also in glial cells. In consistent with reports by Delay‐Goyet et al. (2016); Masini et al. (2017), we found that APP/PS1 Tg mice showed memory impairment and could be rescued by administration of thioperamide. Aβ is thought to play an essential pathogenic role in AD, and its deposition in the brain is the initiating step of AD pathogenesis, leading to subsequent neuron and synaptic loss, and cognitive decline (Hardy, 2006; Hardy & Higgins, 1992; Long & Holtzman, 2019). Therefore, we examined the neuronal loss *in vivo* and *in vitro*. As expect, our results suggested that thioperamide improved the decreased number of neuron in either hippocampus or cortex region in APP/PS1 Tg mice *in vivo*. In addition, the *in vitro* study also suggested that thioperamide protected against Aβ‐induced neurotoxicity in primary neurons. A key enzyme involved in the generation of Aβ is the BACE1, for which powerful inhibitors could rescue the functional neuronal impairments in AD (Ghosh & Osswald, 2014; Keskin et al., 2017; Peters et al., 2018; Liu et al., 2020). In this study, thioperamide reduced the Aβ plaque burden and inhibited the expression of BACE1 in APP/PS1 Tg mice. During clinical trial, some inhibitors were well tolerated while others had to stop due to toxicity (Vassar, 2014). Our study showed that thioperamide had a long‐term effect of BACE1 inhibition, showing a well tolerability. Therefore, this study suggested that thioperamide could improve the cognitive impairment, reduce the neuronal loss, and decrease the cerebral Aβ pathology of AD.

The above results indicated that thioperamide alleviated cognitive dysfunction and Aβ pathology in APP/PS1 Tg mice. However, the mechanism remained to be studied. Multiple lines of evidence support the hypothesis that autophagy dysfunction plays an important role in AD pathophysiology (Cheng et al., 2018; Di Meco et al., 2020). The defective autophagy in AD is mainly attributed to either lowered transcription of autophagy‐related genes, which induces impairment of autophagy production or lysosomal proteolytic failure, which results in the intraneuronal accumulation of autophagic vacuoles (Boland et al., 2018; Pickford et al., 2008). AMPK‐mediated autophagy induction by resveratrol activates autophagy and intracellular clearance of Aβ by the lysosomal pathway (Vingtdeux et al., 2010). Suppression of mTOR signaling by rapamycin also leads to an increase in autophagy induction and reduced Aβ deposits and rescued memory deficits in animal model of AD (Caccamo et al., 2014). On the early stage of AD, autophagy can accelerate the clearance of denatured protein and promote the survival of neurons. With the development of AD, the capacity of clearing protein aggregates is decreased. Therefore, it is of great scientific significance to study the dynamic changes of autophagy flux in the pathogenesis of AD and to find new targets for the prevention and treatment of AD (Zhao et al., 2020). Our previous study indicated that thioperamide activated autophagy under cerebral ischemic injury (Yan et al., 2014). Thus, we further explored the effect of thioperamide on autophagy in AD *in vivo* and *in vitro*. In contrast with WT mice, APP/PS1 Tg mice exhibited either less autophagic level or autophagic flux in hippocampus and cortex, and both of them could be rescued by administration of thioperamide. Consistent with the *in vivo* results, with optimum concentration of 10^−6^ M applied on primary neurons, thioperamide could up‐regulate the autophagic puncta and autophagic flux in primary neurons under Aβ‐induced injury. Protein P62 is an autophagic cargo receptor binding directly to LC3 (Liu & Li, 2019; Pankiv et al., 2007). A decrease of P62 often accompanies accelerated autophagy, while accumulation may indicate a decrease (Boland et al., 2018). We observed a significant accumulation of P62 expression in APP/PS1 Tg mice, which was reversed significantly by thioperamide in either hippocampus or cortex. Consistent with the *in vivo* study, we also found that the increased expression of P62 was reversed significantly by thioperamide in primary neurons against Aβ‐induced injury, suggesting that thioperamide enhanced the impaired production of autophagy. Our results were consistent with reports by Zhao et al. that decreased levels of autophagic‐related genes and autophagic flux were examined in 9‐month‐old and 12‐month‐old transgenic AD mice (Manczak et al., 2018; Zhao et al., 2020). However, it is controversial because some studies indicate an increased autophagic level in 4‐ to 6‐month‐old AD mice (Long et al., 2020; Sanchez‐Varo et al., 2012). The differences of these observations might be attributed to the age‐dependent down‐regulated expression of autophagy‐related genes following the pathology progression in AD (Omata et al., 2014). The increased expression autophagic‐related protein levels in APP/PS1 Tg mice was only evidenced at early ages (4–6 months) (Sanchez‐Varo et al., 2012). However, at the moderate to the later stage of AD, elevated oxidative stress induces age‐dependent reduction of autophagy‐related gene expression (Li et al., 2017; Vegh et al., 2019).

In addition, we also observed an increase of autophagic cells near plaque by thioperamide. Reports show that Aβ could be degraded by autophagy through the autophagy receptor optineurin in microglia (Cho et al., 2014). Except for microglia, neuronal mitophagy also contributes to rescue synaptic dysfunction and cognitive deficits by degrading Aβ and Tau accumulation through decreases in oxidative damage and cellular energy deficits (Kerr et al., 2017). Up‐regulation of autophagy in primary neurons and increased autophagic cells around plaque by thioperamide suggested that thioperamide might stimulate neuronal mitophagy, prevent mitochondrial damage, and trigger elimination of Aβ containing plaque in AD.

Enhanced autophagy improves learning behavior and reduces the accumulation of Aβ and BACE1 degradation (Alam & Scheper, 2016; Feng et al., 2017; Steele et al., 2013; Yang et al., 2011a). Consistent with these results, our results showed that autophagic inhibitor 3‐MA could block the improvement effect of thioperamide on cognitive function, suggesting that thioperamide improved the cognitive deficit in APP/PS1 Tg mice through up‐regulation of autophagic level. Our *in vitro* study also showed that the alleviated neurotoxicity on primary neurons against Aβ was reversed significantly by inhibition of autophagy by either 3‐MA or siRNA of *Atg7*, suggesting thioperamide protected primary neurons against Aβ‐induced injury via improving autophagy. Moreover, the effect of thioperamide on decreased deposition of Aβ and accumulation of BACE1 in either hippocampus or cortex was reversed by inhibition of autophagy by administration of 3‐MA, suggesting that improved effect of thioperamide on Aβ pathology was related to up‐regulation of autophagy. Above all, these results indicated that enhancement of autophagy by thioperamide contributed to the alleviated effect of thioperamide on cognition and Aβ pathology.

Reduced p‐CREB level has been observed in Tg‐AD mice overexpressing Aβ (Dineley et al., 2001). Therefore, deficit in CREB signaling may be implicated in AD pathology through the detrimental effects of Aβ (Scott Bitner 2012; Bartolotti et al., 2016; Yin et al., 2016). The decreased p‐CREB expression is involved in the impaired autophagy in AD mice (Chong et al., 2018). Up‐regulation of p‐CREB activates autophagy and protects against Aβ‐induced injury (Singh, Bissoyi, et al., 2017; Singh, Kashyap, et al., 2017). Implicating CREB, a downstream signaling of H3R (Leurs et al., 2005), might play an important role in mediating the effect of thioperamide on autophagy. Therefore, we investigated the effect of thioperamide on CREB phosphorylation. As expect, decreased level of p‐CREB was observed in Aβ‐induced injury in primary neurons, which was rescued significantly by administration of thioperamide. H89, an inhibitor of protein kinase A (PKA)/CREB, reverses the alleviated effect of thioperamide on cell viability in primary neurons, suggesting that thioperamide protected against Aβ‐induced injury through activating CREB pathway. Both elevated autophagic level and autophagic flux by thioperamide were also reversed by administration of H89 in primary neurons, indicating that thioperamide up‐regulated autophagy through activating CREB signaling.

Both CREB overexpression and activation induces up‐regulation of autophagy‐related protein Atg7 and lysosomal‐related proteins TFEB and LAMP1 (Chong et al., 2018; Seok et al., 2014). Atg7 up‐regulation induces reduction of Aβ‐mediated cytotoxicity in primary neurons (Wani et al., 2019; Wei et al., 2019). Overexpression of TFEB induces increased level of LAMP1 and facilitates autophagy‐lysosomal pathway to influence the process of amyloid β‐protein (Aβ) generation in neurons (Yamamoto et al., 2019). In order to investigate the mechanism of up‐regulation of autophagy mediated by CREB activation by thioperamide, we further studied whether induction of autophagy by thioperamide was accompanied by up‐regulation of these autophagy‐related proteins and lysosomal proteins. As expect, our results showed that thioperamide up‐regulated either the autophagy‐related protein Atg7 or lysosomal‐related proteins, including TFEB and LAMP1. In support of our studies, reports show that TFEB transfection stimulates lysosome biogenesis and attenuate Aβ generation by accelerating flux through the endosome‐lysosome pathway (Xiao et al., 2015), suggesting that targeting TFEB activation could accelerate removal of Aβ deposits as a veritable arm of a multipronged approach to the treatment of AD. Interestingly, the up‐regulated proteins offered by thioperamide were all compromised with CREB inactivation by H89, suggesting that the activation of autophagic function by thioperamide was dependent of CREB‐mediated increased expression of Atg7, TFEB, and LAMP1. Indeed, BACE1 are also degraded within lysosomes (Chen et al., 2000; Koh et al., 2005), suggesting that lysosomes may be the critical determinant of whether APP metabolism leads to Aβ generation or its non‐amyloidogenic degradation. Importantly, our study showed that CREB activation was also involved in the regulation of amyloidogenic cleavage of APP, because H89 reversed the expression of BACE1 expression. CREB activation could regulate autophagy/lysosomal genes expression by up‐regulating TFEB (Settembre et al., 2011, 2013). Some autophagic‐related genes could to be regulated by either CREB or TFEB or by both (Seok et al., 2014). Therefore, activation of CREB and subsequent expression of autophagy/lysosomal protein is critical to the degradation of BACE1 and inhibition of amyloid pathological process in AD.

**FIGURE 9:**
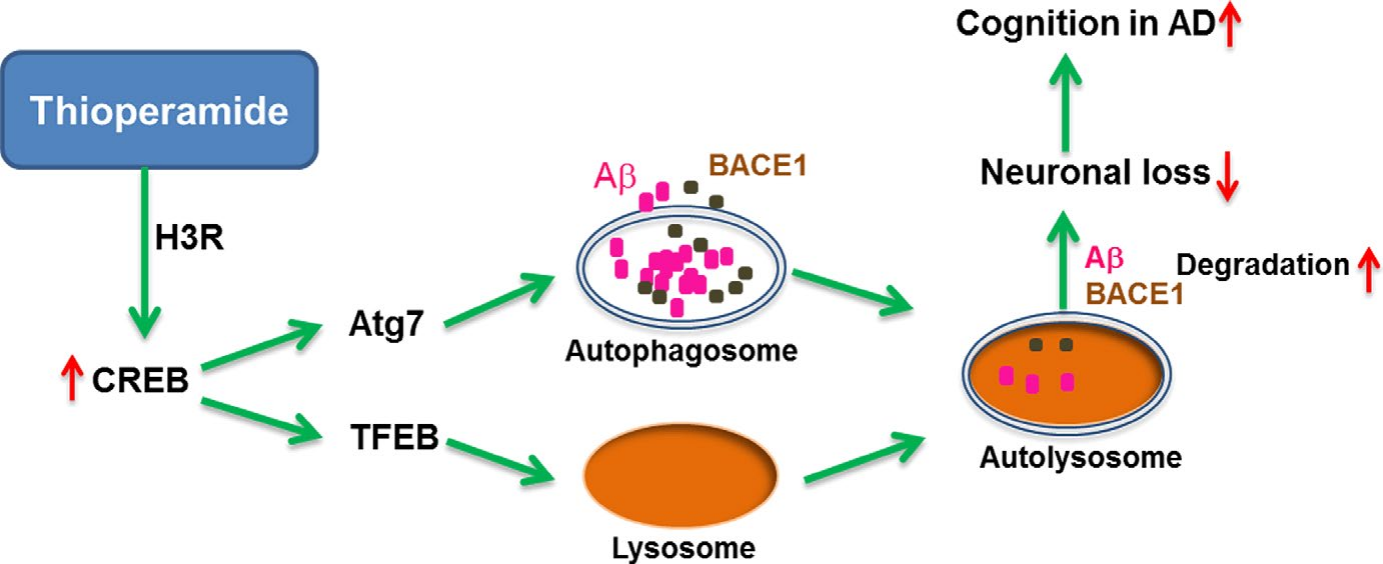
A proposed model for the neuroprotective effects of thioperamide against AD‐like pathology. Inhibition of H3R by thioperamide activates CREB, upregulates expression of Atg7 and TFEB and activates autophagy, then induces removal of BACE1 and Aβ and reverses cognitive deficits in AD

In conclusion, we demonstrated that the H3R antagonist thioperamide improved cognitive impairment in APP/PS1 mice via modulation of the CREB‐mediated autophagic and lysosomal pathway, which contributed to Aβ clearance (Figure 9). These results uncovered a novel mechanism behind the therapeutic effect of thioperamide in AD and further provided an experimental basis for starting a clinical trial for H3R antagonists as a treatment for AD.

## 4 MATERIALS AND METHODS

### 4.1 Animals

Adult male wild type (WT) and BL/6‐Tg (APPswe, PSEN1dE9) (APP/PS1) mice of 8 months (from Jackson Lab, Stock Number: 004462) were used in this study. The mice were housed in a temperature‐ and humidity‐controlled animal facility, which was maintained on a 12 h light/dark cycle, food and water were given *ad libitum*. All animal studies were carried out according to protocols approved by the Institutional Animal Care and Use Committee of Binzhou Medical University Hospital and conducted in compliance with the National Institutes of Health Guide for the Care and Use of Laboratory Animals. Efforts were made to minimize any pain or discomfort, and the minimum number of animals was used.

### 4.2 Stereotaxic surgery

Stereotaxic surgery was performed as previously described (Liu et al., 2020). Mice were anesthetized with the intraperitoneal injection of 1% chloral hydrate and then immobilized on a stereotactic frame. The gauge guide cannula was implanted into the lateral ventricle (0.2 mm posterior, 1.1 mm lateral, and 2.7 mm ventral to the bregma). After surgery, mice were housed individually and allowed to recover for 7 day.

### 4.3 Drug treatments

For *in vivo* study, a stainless‐steel injector connected to a 5‐μl syringe was inserted into the guide cannula and extended 1 mm beyond the tip. The administration of the chemicals was administrated on the basis of previous studies (Yan et al., 2014). Thioperamide (i.p., 5 mg/kg) or vehicle was administrated daily at 7 days after stereotaxic surgery until the beginning of the behavior tests on day 14. 3‐MA or vehicle (i.c.v., 15 µg in 2 µl vehicle) was administrated 0.5 h before thioperamide injection. Chloroquine (i.p. 60 mg/kg) was administrated 0.5 h before thioperamide injection. Thioperamide and chloroquine were dissolved in saline, and 3‐MA was dissolved dimethyl sulfoxide. At 14 days after stereotaxic surgery, the novel object recognition (NOR) test was carried on for 4 days. At 18 days after stereotaxic surgery, Y maze (YM) test was carried on for 1 day. At 19 days after stereotaxic surgery, morris water maze (MWM) test was carried on for 6 days. During the period of behavior test, thioperamide or vehicle was administrated 0.5 h, whereas 3‐MA or vehicle was administrated 1 h before the test at day 14, day 18, and day 19.

For *in vitro* study, the indicated concentrations of Aβ_1–42_ (obtained by incubating Aβ_1–42_ for 24 h at 4°C, Millipore), thioperamide (Abcam), and chloroquine (Sigma) were dissolved in PBS, 3‐MA (Sigma) and H89 (Sigma) were dissolved in dimethyl sulfoxide. Thioperamide was administrated 0.5 h before Aβ_1–42_ administration, whereas 3‐MA (2.5 mM), chloroquine (10 μM), and H89 (10 μM) were administrated 1 h before Aβ_1–42_ treatment. The indicated concentrations of the chemicals were administrated on the basis of previous studies (Yan et al., 2014).

### 4.4 Novel object recognition (NOR)

The NOR test was performed 14 days after stereotaxic surgery as previously described (Chiba et al., 2009). Briefly, mice received 2 d of habituation in a 45 × 45 cm square arena, and on the third day, they were allowed to explore two identical objects made from large Lego bricks for 10 min (training trial). They were returned to their home cage, and 24 h later, a different shape and color object replaced one of the objects and the mice were returned to the arena for 10 min (testing trial). The time spent on each object was then calculated as a percentage of total object exploration.

### 4.5 Y maze (YM)

The Y maze test was performed 18 days after stereotaxic surgery as previously described (Chiba et al., 2009). Briefly, the apparatus for YM was made of gray plastic, with each arm 40 cm long, 12 cm high, 3 cm wide at the bottom, and 10 cm wide at the top. The three arms were connected at an angle of 120°. Mice were individually placed at the end of an arm and allowed to explore the maze freely for 8 min. The total arm entries and spontaneous alternation percentage (SA %) were measured. SA % was defined as a ratio of the arm choices that differed from the previous two choices (“successful choices”) to total choices during the run (“total entry minus two” because the first two entries could not be evaluated). For example, if a mouse made 10 entries, such as 1–2–3–2–3–1–2–3–2–1, there are 5 successful choices in 8 total choices (10 entries minus 2). Therefore, SA% in this case is 62.5%.

### 4.6 Morris water maze (MWM)

The MWM maze test was performed 19 days after stereotaxic surgery as previously described (Vorhees & Williams, 2006). Briefly, the water maze of 1.50 m in diameter and 0.50 m in height was filled with water (20 ± 1°C) to maintain the water surface 1.50 cm higher than the platform (10 cm in diameter). Water was dyed white and the tank was divided into four quadrants by four points: North (N), South (S), East (E), and West (W). The platform was placed at the center of either quadrant and video tracking software was used to automatically track the animals. Learning and memory acquisition lasts for five days. Animals were put into the water from four points in random order every day until they found the platform and stayed for 10 s within 1 min. If the mice cannot find the platform within 1 min, they were guided to the platform. Following acquisition test, on the sixth day, learning and memory maintenance test was carried on. The platform was removed, and the mice were placed in water from the opposite quadrant of the platform, and then the times crossing the platform was recorded within 1 min.

### 4.7 Immunohistochemistry

Immunostaining was performed in cultured neurons and frozen brain sections. Neurons seeded on coverslips were fixed in cold methanol for 10 min and frozen brain sections were fixed in 4% paraformaldehyde for 2 h, and then incubated in 5% bovine serum albumin (BSA, Solarbio) for 2 h to block nonspecific binding of IgG. Then, the cells were reacted with rabbit monoclonal antibody against mouse monoclonal antibody against NeuN (1:300; MAB377, Millipore), rabbit polyclonal antibody against LC3B (1:100, abcam), mouse monoclonal antibody against H3R (1:50, Santa Cruz), and β‐Amyloid (1:100, abcam). After repeated washes in PBS buffer, cells were incubated with secondary antibody in 3% BSA for 2 h at 25°C. The secondary antibodies used in this experiment were goat anti‐mouse IgG‐AlexaFluo 488 (1:300, Invitrogen), goat anti‐rabbit IgG‐AlexaFluo 594 (1:300, Invitrogen), and goat anti‐rabbit IgG‐AlexaFluo 488 (1:300, Invitrogen). After further washing in PBS, cultures were dried, cover slipped and mounted on glass slides. For thioflavin‐S staining, sections were incubated with 70 and 80% ethanol and stained with 1% thioflavin S (Sigma) in 80% ethanol for 15 min. Afterward, sections were rinsed in 80% and 70% ethanol and distilled water. The stained cells were observed under a laser scanning confocal microscope (Leica TCS SPE, Germany). All the immunohistochemical data represent mean values of 5 brain sections per mouse. The plaque and Aβ area coverage as well as the number of NeuN‐positive neurons, LC3‐positive autophagic cells were quantified using ImageJ. The number of near‐plaque LC3‐positive autophagic cells was defined as near plaque when they were located ≤50 μm around plaques.

### 4.8 Western blot

Mice were anesthetized by i.p. injection of chloral hydrate (400 mg/kg), sacrificed and the brain was quickly removed. The separated tissue, primary neurons were lysed in ice‐cold RIPA lysis buffer (R0020, Solarbio), then centrifuged at 14 000***g*** at 4°C for 20 min, and the protein concentration in the extracts was determined by the Bradford assay (Thermo, Hercules, CA). The precipitates were denatured with SDS sample loading buffer and separated on 10% SDS‐PAGE. Proteins were transferred onto nitrocellulose membranes using a Bio‐Rad mini‐protein‐III wet transfer unit overnight at 4°C. Transfer membranes were then incubated with blocking solution (5% nonfat dried milk dissolved in tris buffered saline tween (TBST) buffer (in mM): 10 Tris‐HCl, 150 NaCl, and 0.1% Tween‐20) for 2 h at room temperature, and incubated with primary antibody overnight at 4°C. The primary antibodies used in this experiment were BACE1 (ab2077, abcam, 1:1000), LC3 (PM036, MBL, 1:1000), P62 (ab109012, abcam, 1:1000), Atg7 (ab133528, abcam, 1:1000), phospho‐CREB (9198, Cell Signaling Technology, 1:1000), CREB (9197, Cell Signaling Technology, 1:1000), TFEB (ab264421, abcam, 1:1000), LAMP1 (ab24170, abcam, 1:1000), and GAPDH (KC‐5G4, Kangchen Biotech, 1:3000). Membranes were washed three times in TBST buffer and incubated with the appropriate secondary antibodies (LI‐COR, Odyssey, 1:5000) for 2 h. Images were acquired with the Odyssey infrared imaging system and analyzed as specified in the Odyssey software manual. The results were expressed as the target protein/GAPDH ratio and then normalized to the values measured in the control groups (presented as 100%).

### 4.9 Cell culture

For primary neuronal cell culture, pregnant C57BL6J mice were anesthetized by intraperitoneal injection of 1% chloral hydrate (Klontech), and the cortex was isolated from embryos (18 days). Cells (800–1000 cells/mm^2^) were seeded on coverslips coated with 30 mg/ml poly‐d‐lysine (P1399, Sigma). Cells were placed in fresh serum‐free neurobasal medium (21103, Gibco) plus 2% B27 and fed every 4 days with fresh medium and used after 7 days (DIV7). The cells were incubated at 37°C in a 5% CO_2_ humidified atmosphere.

### 4.10 Cell viability assay by MTT

The viability of primary neurons was measured by 3‐(4,5‐dimethylthiazol‐2‐yl)‐ 2,5‐diphenyltetrazolium bromide (MTT, Sigma) assay. Briefly, cells were incubated with 0.5 mg/ml MTT for 2 h, the supernatant layer was then removed, and 100 µl/well of dimethyl sulfoxide (DMSO, Solarbio) was added into the 96‐well plates. MTT metabolism was quantitated spectrophotometrically at 570 nm in a Bio‐Rad microplate reader. Results were expressed as the percentage of MTT reduction, taking the absorbance of control cells as 100%.

### 4.11 RNA interference

Small‐interfering RNA (siRNA) targeting mouse Atg7 were synthesized by corporation (GenePharm, Shanghai) as follows: negative control, AUGAACGTGAAUUGCUCAA; Atg7, GCAUCAUCUUUGAAGUGAA. Primary neurons on DIV5 were transfected with 20 nmol siRNA of negative control and Atg7 using Lipofectamine RNAiMAX (Invitrogen). After transfection in antibiotic‐free medium for 8 h, cells were refreshed with normal medium. Experiments were performed 72 h after transfection.

### 4.12 RT‐PCR

Total RNA from mice hippocampus and cortex was extracted using Trizol reagent (Invitrogen, USA). Reverse transcription was performed with an Exscript RT Reagent Kit (Takara Bio Inc., China). Real‐time PCR analysis was undertaken using SYBR Premix Ex Taq (Takara Bio Inc., China). The primer sequences for TFEB were 5′‐GCCATGCTACATATCAGCTCCAA‐3′ (sense) and 5′‐ATGTAGCCCAGCACGCTGTC‐3′ (antisense). The primer sequences for Atg7 were 5′‐ATGGGAACCCTGCACAACAC‐3′ (sense) and 5′‐TTCGAGAGCAGCACCTGACTTTA‐3′ (antisense). The primer sequences for p62 were 5′‐CCTTGCCCTACAGCTGAGTC‐3′ (sense) and 5′‐TGTTCCACATCAATGTCAACCT‐3′ (antisense). The primer sequences for β‐actin were 5′‐AGCCATGTACGTAGCCATCC‐3′ (sense) and 5′‐TGTGGTGGTGAAGCTGTAGC‐3′ (antisense). Polymerase chain reaction parameters were 95°C for 3 min, and 40 cycles of 95°C for 15 s and 60°C for 1 min. The relative expression of the genes was normalized to the mean levels of β‐actin.

### 4.13 Statistical analysis

Results are expressed as mean ± SEM. Statistical analysis was performed by one‐way ANOVAs followed by Tukey's *post hoc* comparisons or two‐way ANOVAs followed by Bonferroni *post hoc* comparisons, using prism software. *P* value <0.05 was considered statistically significant.

## CONFLICT OF INTEREST

The authors declare no conflict of interest.

## AUTHOR CONTRIBUTIONS

H.J.Y. conceived and designed the project. H.J.Y., J.G.W., B.L., Y.X., M.Z.Y., and C.Y.W., M.M.S., J.L., W.T.W., J.J.Y., F.J.S., D.W., and D.J.L. performed the experiments. H.J.Y., J.G.W., and B.L. analyzed the data and drafted the manuscript.

## Supporting information

Fig S1

Fig S2

Supplementary Material

## Data Availability

The data that support the findings of this study are available from the corresponding author upon reasonable request.
