# Supplementary material for: Activation of CREB‐mediated autophagy by thioperamide ameliorates β‐amyloid pathology and cognition in Alzheimer’s disease
Source: Aging Cell. 2021 Mar 8;20(3):e13333. doi: 10.1111/acel.13333 (PMC7963336; doi:10.1111/acel.13333)
Supplement: Supplementary file 3 — Supplementary Material [file ACEL-20-e13333-s003.docx]

**Supplementary Fig. 1. Expression of H3R in the brain.** (A) Representative images showing the staining of H3R (green) in either hippocampus, including CA1, CA3 and DG or cortex in mice brain. Scale bar: 100 μm (upper panel); 20 μm (lower panel). (B) The expression of H3R is examined by western blot in primary neurons, microglia and astrocytes. n = 4 per group. ****P* <0.001. Mean ± SEM. One-way ANOVA followed by Tukey’s *post hoc* test.

**Supplementary Fig. 2. The long-term effect of thioperamide on BACE1 inhibition.** (A-B) Representative Western blots and bar graph showing the effects of thioperamide on BACE1 expression in either hippocampus (A) or cortex (B) in WT and APP/PS1 mice 3 months after drug treatment. n = 4 per group. ***P* <0.01, ****P* <0.001 *vs*. the vehicle group. Mean ± SEM. One-way ANOVA followed by Tukey’s *post hoc* test.
